# Effect of salicylic acid on expression level of genes related with isoprenoid pathway in centella (*Centella asiatica* (L.) Urban) cells

**DOI:** 10.1007/s13205-016-0404-z

**Published:** 2016-03-05

**Authors:** Nguyen Hoang Loc, Nguyen Thanh Giang, Nguyen Duc Huy

**Affiliations:** 1 College of Sciences, Hue University, Hue, 530000 Vietnam; 2 Institute of Biotechnology, Hue University, Hue, 530000 Vietnam

**Keywords:** *CabAS*, *CaCYS*, *CaSQS*, *Centella asiatica*, Centelloside, Elicitor, Salicylic acid

## Abstract

In this study, we report the expression level of *CaSQS*, *CabAS* and *CaCYS*, the genes involved in phytosterol and triterpene metabolic pathway of centella (*Centella asiatica* (L.) Urban), in cells elicited with salicylic acid (50–200 µM). Reverse transcription-polymerase chain reaction (RT-PCR) and Northern blot analysis indicated *CaSQS*, *CabAS*, and *CaCYS* genes expressed in both the wild-type and cultured cells (with and without elicitation). In elicited cells, expressions of *CaSQS*, *CabAS*, and *CaCYS* genes showed strong dependence on salicylic acid concentration and elicitation day. The highest expression of *CabAS* gene was found in the cells elicited with 100 µM salicylic acid on day 10 of inoculation. Salicylic acid treatment (50–200 µM) decreased expression level of *CaCYS* and *CaSQS* genes in elicited cells compared with the control.

## Introduction

Centella (*Centella asiatica* (L.) Urban) is a perennial, faintly aromatic and a valuable medicinal herb. It is widely distributed throughout tropical and subtropical regions of the world (Seevaratnam et al. 2012). Centella has been used as a traditional herbal medicine in many Asian countries for hundreds of years (Brinkhaus et al. 2000).

Triterpene saponins are a class of plant secondary metabolites with structure derived from the precursor oxidosqualene in which one or more sugar residues are added (Yendo et al. 2010). Triterpene saponins in centella mainly include centellosides (asiaticoside, asiatic acid, madecassoside and madecassic acid) (Bylka et al. 2013). Extract of centella contains centellosides that can elevate antioxidant level in healing wounds, increasing fibroblast production, collagen formation and angiogenesis (Li et al. 2007; Shukla et al. 1999; Maquart et al. 1999). These components are also known to be clinically effective on systemic scleroderma, abnormal scar formation, and keloids (Hong et al. 2005). Although the centellosides have many important pharmaceutical properties, their content is not significant in plant, thus it is difficult to scale up production. Plant cell cultures were, therefore, widely used as a convenient tool to provide a valuable alternative for the production of important secondary metabolites for commercial interest.

**Fig. 1:**
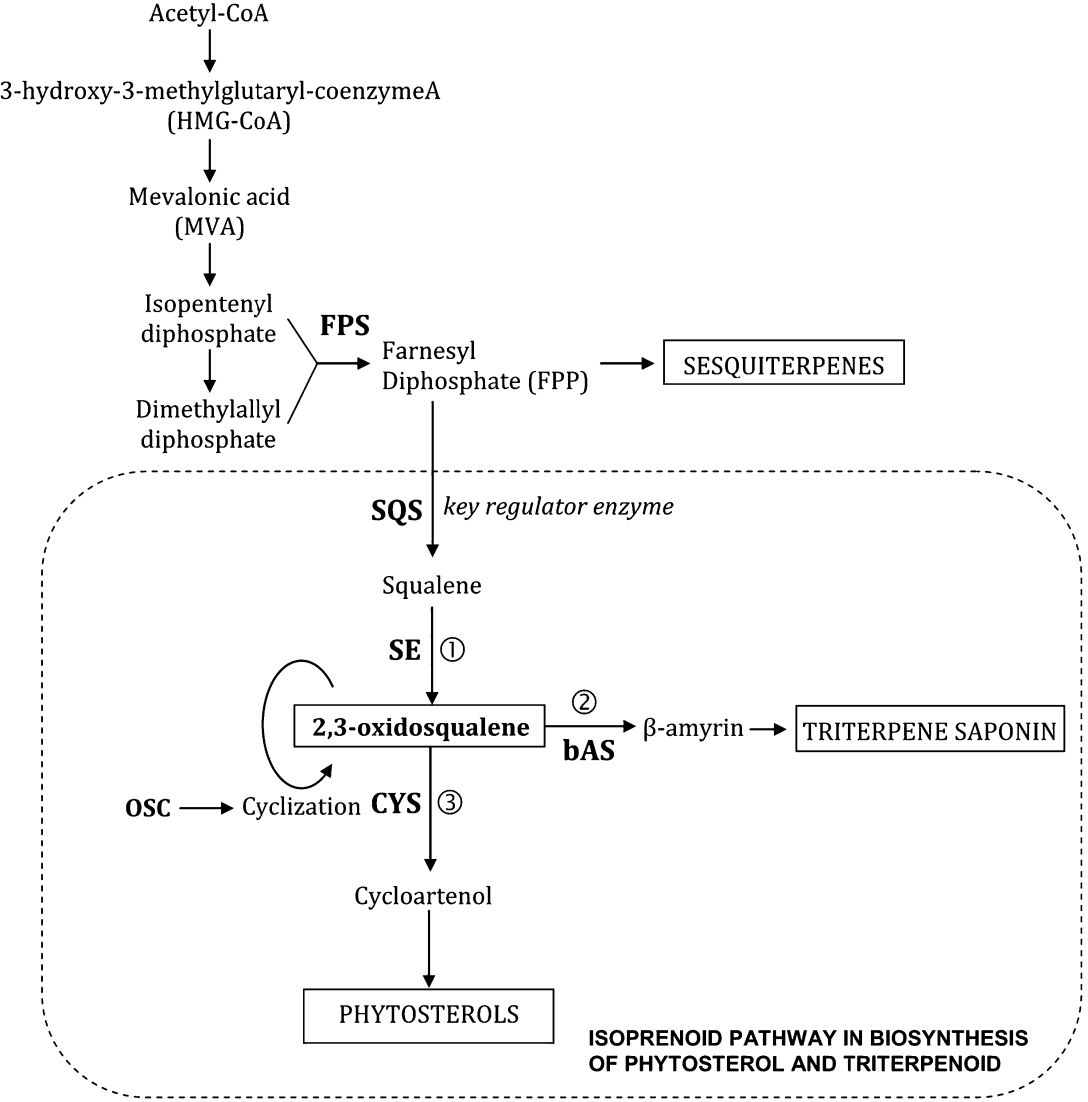
Isoprenoid pathway in biosynthesis of phytosterol and triterpenoid in centella (Kim et al. 2005a, b, c, 2009; Mangas et al. 2008). *FPS* farnesyl diphosphate synthase, *SE* squalene epoxidase, *OSC* oxidosqualene cyclase

There were some reports on the biosynthesis of centellosides and phytosterol from in vitro cultures of centella. These studies investigated into the effects of methyl jasmonate (MeJA), as an elicitor, in relation to expression levels of genes that participate in triterpene metabolism (isoprenoid pathway) in cultured centella cells such as *CaSQS* (*Centella asiatica* squalene synthase), *CabAS* (*C. asiatica* β-amyrin synthase), and *CaCYS* (*C. asiatica* cycloartenol synthase) (Fig. 1) (Kim et al. 2005a, b, c, 2009; Mangas et al. 2008). *CaSQS* and *CabAS* are two genes that produce large quantities of triterpene saponins such as asiaticoside and madecassoside, in which *CaSQS* is considered a key regulator gene. *CaCYS* gene codes cycloartenol synthase, the enzyme responsible for the first step in sterol biosynthesis (Mangas et al. 2006).

According to Jirage et al. (1999), salicylic acid is an important signal molecule, it activate genes related to plant protection against pathogenesis. When used as an elicitor, salicylic acid is very useful for the accumulation of the bioactive compounds relate to pathogenesis. However, there was no report on the effect of salicylic acid on the biosynthesis of centellosides, and the relationship between salicylic acid elicitation and metabolic genes in cultured cells. While this research direction was performed in some other plant species, for example Yu et al. (2006) found the relationship between expression levels of *chs* (chalcone synthase) and *chi* (chalcone isomerase) genes with contents of jaceosidin and syringin in *Saussurea medusa* cells treated with salicylic acid. Yousefzadi et al. (2010) found salicylic acid elicitation increased expression levels of the genes coding for phenylalanine ammonia-lyase, cinnamoyl-CoA reductase and cinnamyl-alcohol dehydrogenase in the first steps of podophyllotoxin pathway in *Linum album*. However, expression of the pinoresinol–lariciresinol reductase gene, which is involved in one of the last biosynthetic steps, was not affected by salicylic acid.

Our previous studies have demonstrated that an optimal concentration of salicylic acid (100 µM) can stimulate asiaticoside production, a major component of centelloside, in centella cells up to 229.83 mg/g dry weight, while an optimal concentration of MeJA (100 µM) or yeast extract (4 g/L) only enhances asiaticoside production to 205.92 or 165.41 mg/g dry weight, respectively (Loc and Giang 2012; Giang et al. 2015). So the present work was setup based on this idea to find the molecular mechanism of gene regulation under the influence of salicylic acid. The results may provide us with a clearer understanding of problems related to salicylic acid elicitation to improve the productivity of the centelloside biosynthesis in cultured cells of centella. The effect of salicylic acid on gene regulation in the isoprenoid pathway can be applied to control the biotechnological production of centelloside.

## Materials and methods

### Plant materials

Centella suspension cells were cultured as our previous report (Loc and Giang 2012). Three grams of cells were inoculated in 250-mL Erlenmeyer flask containing 50 mL of nutrient medium and incubated at 25 ± 2 °C on the rotary shaker with a speed of 120 rpm for 24 days under an intensity of 360 lux to produce biomass.

Elicitation effect of salicylic acid was studied by adding different concentrations (50–200 μM) to the medium at the beginning of cell culture, and days 5, 10 and 15 after inoculation. The cell biomass was harvested after 24 days by filtration, expression level of *CaSQS, CabAS* and *CaCYS* genes was analyzed by reverse transcription-polymerase chain reaction (RT-PCR) and Northern blot.

### cDNA synthesis and preparation of probe

Total RNA was isolated from centella 14-day-old cells using the Invitrap^®^ Spin Plant RNA Mini Kit (Stratec Molecular GmbH, Berlin, Germany) according to the manufacturer’s instructions.

First strand cDNA was synthesized by the First Strand cDNA Synthesis Kit (#K1612, Fermentas) in a final volume of 20 µL with 5 µg of total RNA, 0.5 µg of oligo(dT)_18_ primer, 4 µL of 5× reaction buffer, 20 unit of RiboLock^TM^ ribonuclease inhibitor, 2 µL of 10 mM dNTP mix and 40 units of M-MuLV (Moloney-murine leukemia virus) reverse transcriptase. The mixture was incubated at 37 °C for 60 min, stopped at 70 °C for 5 min and kept at 4 °C in ice bath.

The probes for *CaSQS, CabAS* and *CaCYS* were described by Bonfill et al. (2011). The primer sequences corresponding to the probes are listed in Table 1. The PCR amplifications for probes were performed in a thermal cycler (MyCycler^TM^, Bio-Rad, USA) using PCR master mix (#M7502 Promega, Madison, USA). The PCR mixture consisted of 125 ng cDNA, 6 µL of 2× master mix, 10 pmol of each primer, and double distilled water to a final volume of 12 µL. All the PCRs were carried out under the following conditions: genomic denaturation at 95 °C for 5 min, followed by 30 cycles at 95 °C for 30 s, 55 °C for 30 s and 72 °C for 30 s, and a final extension at 72 °C for 10 min. PCR amplicons were then purified and inserted into pGEM^®^-T Easy vector (Promega, Madison, USA) and transformed into *E. coli* TOP10 cells. The nucleotide sequence of the insert was confirmed by the method of fluorescent dideoxy-terminator on 3130 ABI system (Applied Biosystem).

**Table 1:** Oligonucleotide primers used in the PCR amplification for the probes of *CaSQS, CaCYS* and *CabAS* genes (Bonfill et al. 2011)

No.	Primers	Nucleotide sequences
1	CaSQS-F	5′-TGGGTTAGGGTTGTCAAAGC-3′
	CaSQS-R	5′-CGGAAGATAGCAGGATCTCG-3′
2	CabAS-F	5′-TGGTTGGGGAGAAAGTCTTG-3′
	CabAS-R	5′-ACAAGCGTTTGCGGTACTCT-3′
3	CaCYS-F	5′-GAATCCACGCCATGAAGTCT-3′
	CaCYS-R	5′-ACCACCATGATCCAGAATCC-3′

### RT-PCR

The expression levels of *CaSQS, CabAS* and *CaCYS* genes in centella cells elicited with salicylic acid were determined by RT-PCR. The PCR amplification was carried out as described above. The intensities of PCR products were calculated using Quantity One program (ver. 4.1) of Gel Documentation System (Bio-Rad, Hercules, CA, USA).

### Northern blot

The probes of interested genes were labeled with digoxigenin-dUTP using DIG High Prime DNA Labelling and Detection Starter Kit I (Roche, Mannheim, Germany) according to the manufacturer’s instructions.

Forty micrograms total RNA was size-fractionated by 1.2 % (w/v) agarose gel electrophoresis containing 2.2 M formaldehyde and then transferred to a Hybond-N^+^ (Bio-Rad, Hercules, CA, USA) nylon membrane. The blots were hybridized with cDNA probes of interested genes (*CaSQS, CabAS* and *CaCYS*) labeled with digoxigenin-dUTP at 42 °C overnight. After hybridization, blots were washed and incubated with antibody-conjugated digoxigenin and alkaline phosphatase (1:5000 v/v) at room temperature for 30 min. The 5-bromo-4-chloro-3-indolyl-phosphate (BCIP)/nitroblue tetrazolium (NBT) (Roche, Mannheim, Germany) substrate is used for color development. The intensities of hybridization signals were analyzed using Quantity One program (ver. 4.1) of Gel Documentation System.

### Statistical analysis

The experiments were done in triplicate. The data were analyzed as means followed by one-way ANOVA (Duncan’s test, *p* < 0.05).

## Results

### Expression of *CaSQS* gene

In this study, we investigated *CaSQS* gene expression in cell cultures treated with salicylic acid through the transcription using both the RT-PCR and the Northern blot techniques.

**Fig. 2:**
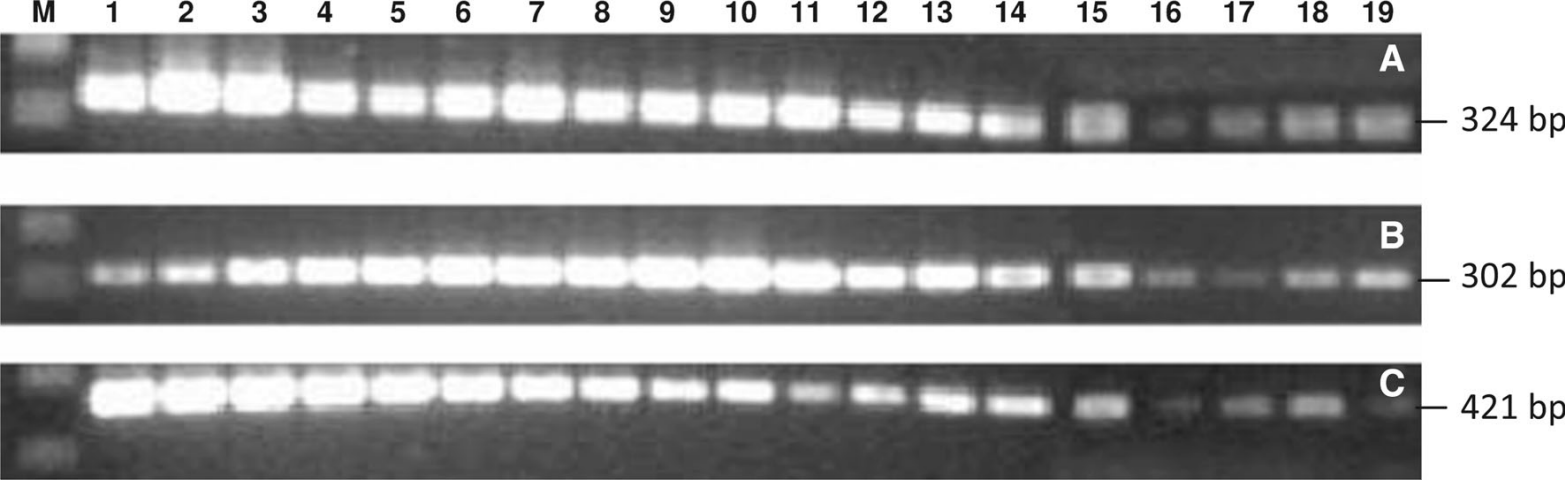
RT-PCR of *CaSQS* (**a**), *CabAS* (**b**) and *CaCYS* (**c**) genes from centella cell cultures. *M*: 100 bp DNA ladder marker; *1* wild-type centella leaf, *2* centella cells at the beginning of culture, *3* centella cells after 24 days of culture, *4*–*7* centella cells treated with 50 µM salicylic acid at the days 0, 5, 10, and 15 after inoculation, respectively, *8*–*11* centella cells treated with 100 µM salicylic acid at the days 0, 5, 10, and 15 after inoculation, respectively, *12*–*15* centella cells treated with 150 µM salicylic acid at the days 0, 5, 10, and 15 after inoculation, respectively, *16*–*19* centella cells treated with 200 µM salicylic acid at the days 0, 5, 10, and 15 after inoculation, respectively. PCR products were separated on 1.5 % agarose gel. Electrophoresis was run at 60 V for 30 min. Images were analyzed using Quantity One program (ver. 4.1) of Gel Documentation System

**Fig. 3:**
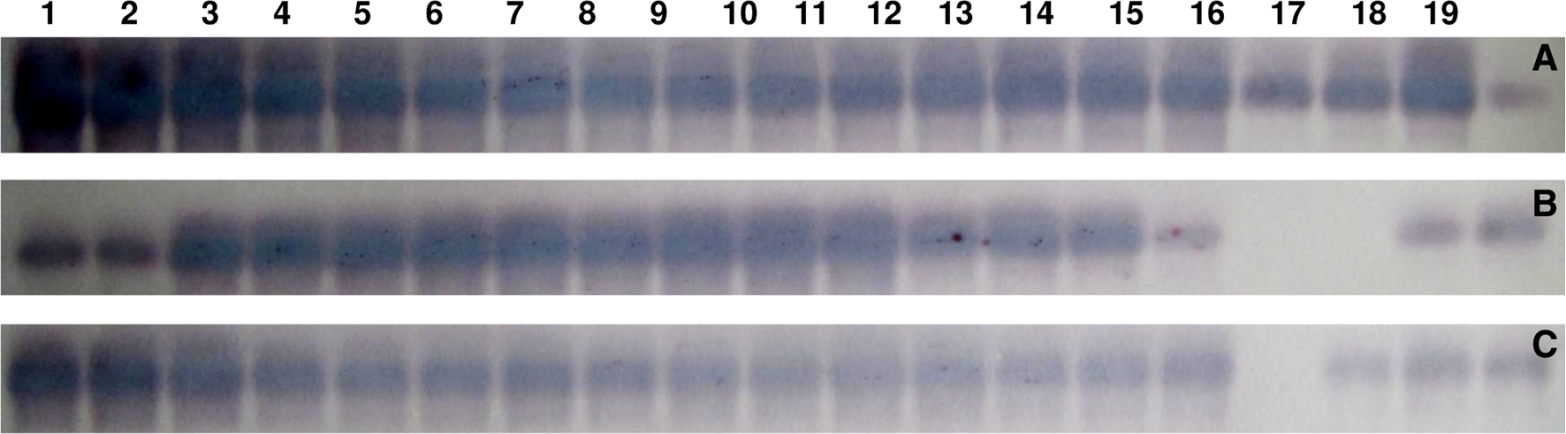
Northern blot analysis of *CaSQS* (**a**), *CabAS* (**b**) and *CaCYS* (**c**) genes from centella cell cultures. *1* wild-type centella leaf, *2* centella cells at the beginning of culture, *3* centella cells after 24 days of culture, *4*–*7* centella cells treated with 50 µM salicylic acid at the days 0, 5, 10, and 15 after inoculation, respectively, *8*–*11* centella cells treated with 100 µM salicylic acid at the days 0, 5, 10, and 15 after inoculation, respectively, *12*–*15* centella cells treated with 150 µM salicylic acid at the days 0, 5, 10, and 15 after inoculation, respectively, *16*–*19* centella cells treated with 200 µM salicylic acid at the days 0, 5, 10, and 15 after inoculation, respectively. Northern blot analysis was carried out as described in materials and methods section. Images were analyzed using Quantity One program (ver. 4.1) of Gel Documentation System

As shown in Fig. 2a, *CaSQS* gene expression was higher in the wild-type leaf and the non-elicited cells than in the elicited cells. Transcript levels of the *CaSQS* gene in elicited cells decreased with increasing salicylic acid concentration or elicitation day, and it seems to be depressed at salicylic concentration of 200 µM during all elicitation times (0–15 days). Similarly, Northern blot analysis revealed stronger hybridization signals in the wild-type and non-elicited cells compared with that in elicited cells (Fig. 3a).

Intensities of DNA bands from RT-PCR and in situ hybridization signals showed they ranged from 401 to 2895 and 443 to 2120, respectively. The highest was found in non-elicited cells at the beginning of culture. A short elicitation time with lower concentration of salicylic acid did not significantly inhibit *CaSQS* gene expression (Table 2).

**Table 2:** Intensities of DNA bands from RT-PCR and in situ hybridization signals from Northern blot of *CaSQS, CabAS* and *CaCYS* genes in centella cells

Gene	Method	Samples																		
		1	2	3	4	5	6	7	8	9	10	11	12	13	14	15	16	17	18	19
*CaSQS*	RT-PCR	2095^c^	2895^a^	2672^b^	1634^e^	1540^f^	1801^d^	2043^c^	1556^f^	1737^de^	1696^de^	1843^d^	1150^h^	1339^g^	1189^h^	1659^e^	401^k^	544^j^	949^i^	1096^h^
	NB	1872^c^	2120^a^	1998^b^	1001^f^	1214^e^	1262^e^	1441^d^	994^f^	1002^f^	1201^e^	1220^e^	845^g^	878^fg^	905^fg^	990^f^	443^j^	689^h^	709^h^	723^h^
*CabAS*	RT-PCR	1200^j^	1432^i^	1986^g^	2256^f^	2592^ce^	2695^c^	2535^e^	2702^c^	3001^b^	4012^a^	2462^e^	1910^g^	2259^f^	1848^gh^	1725^h^	543^l^	501^l^	851^k^	878^k^
	NB	891^fg^	1003^f^	2013^d^	2120^d^	2443^c^	1578^e^	2461^c^	2500^c^	2771^b^	2942^a^	2758^b^	1891^e^	2002^d^	2022^d^	993^f^	443^h^	443^h^	877^fg^	881^fg^
*CaCYS*	RT-PCR	2557^a^	2312^b^	2287^b^	2077^d^	2180^c^	1749^e^	1563^f^	1320^g^	1001	1100^h^	711^j^	843^ij^	1100^h^	1039^h^	903^i^	250^j^	340^k^	320^k^	289^k^
	NB	2696^a^	2356^b^	2122^c^	2005^d^	1987^d^	1673^g^	1886^de^	1921^de^	1890^de^	1802^f^	1920^de^	2117^c^	2011^d^	1935^de^	2000^d^	657^j^	1322^i^	1557^h^	498^k^

Different letters in a row indicate significantly different means (Duncan’s test, *p* < 0.05)

RT-PCR and Northern blot analysis data were recorded and analyzed using Quantity One program (ver. 4.1) of Gel Documentation System

*NB* Northern blot, *1* wild-type centella leaf, *2* centella cells at the beginning of culture, *3* centella cells after 24 days of culture, *4*–*7* centella cells treated with 50 µM salicylic acid at the days 0, 5, 10, and 15 after inoculation, respectively, *8*–*11* centella cells treated with 100 µM salicylic acid at the days 0, 5, 10, and 15 after inoculation, respectively, *12*–*15* centella cells treated with 150 µM salicylic acid at the days 0, 5, 10, and 15 after inoculation, respectively, *16*–*19* centella cells treated with 200 µM salicylic acid at the days 0, 5, 10, and 15 after inoculation, respectively

### Expression of *CabAS* gene

We also analyzed *CabAS* gene expression (coding β-amyrin synthase, the specific oxidosqualene cyclase for centelloside production) in triterpene metabolism of centella cells. RT-PCR results showed *CabAS* gene expression was higher in the elicited cells (100 μM salicylic acid) than in the controls (wild-type and non-elicited cells). The highest levels were achieved on day 10 of inoculation, approximately 3.3- and 2.8-fold higher than the controls, respectively (Fig. 2b; Table 2).

Northern blot analysis also indicated that the cells treated with 100 µM salicylic acid on 10th day of inoculation resulted in the strongest expression of *CabAS* gene (Fig. 3b), the intensity values ranged from 2500 to 2942, approximately 3.3- and 2.9-fold higher than the controls, respectively (Table 2). These results are in accordance with RT-PCR analysis. Expression of *CabAS* gene obviously decreased in the cells treated with higher concentrations (150–200 μM) of salicylic acid.

### Expression of *CaCYS* gene

The effect of salicylic acid elicitation on the expression of *CaCYS* gene was investigated. The RT-PCR and Northern blot analysis indicated that *CaCYS* strongly expressed in the wild-type and non-elicited cells (Figs. 2c, 3c). All concentrations of salicylic acid (50–200 µM) strongly inhibited *CaCYS* gene expression.

Intensity values of RT-PCR and Northern blot analysis ranged from 103 to 2557 and 496 to 2696, respectively. In general, *CaCYS* gene expression in centella cell cultures (with and without elicitation) was weaker than that in the wild type.

Salicylic acid treatment of 100 µM on day 10 of inoculation strongly affected the expression of *CaCYS* gene. The RT-PCR analysis showed that transcription level reduced by 2.5 times compared to the wild type (Figs. 2c, 3c). Similar to the expression of *CaSQS* and *CabAS* genes, centella cells treated with 200 µM salicylic acid also inhibited *CaCYS* gene (Table 2).

## Discussion

*CaSQS, CabAS,* and *CaCYS* genes play important roles in the biosynthesis of phytosterols and triterpenoid in centella. *CabAS* is a key gene for triterpenoid regulation, whereas *CaCYS* gene responds for phytosterols synthesis (Hernandez-Vazquez et al. 2010). In previous report, we showed that salicylic acid elicitation enhanced asiaticoside accumulation, a type of triterpenoid, especially at concentration of 100 µM (Loc and Giang 2012). In present work, we have studied the expression of genes relating to the centelloside biosynthesis under elicitation of salicylic acid (50–200 µM). Our results showed salicylic acid inhibited the expression of *CaSQS* and *CaCYS* genes, while increased the transcription of *CabAS* gene (Fig. 2). Kim et al. (2005b) demonstrated that the expression of *CabAS* in centella leaves was increased along with the accumulation of asiaticoside when elicited by 100 µM MeJA.

Then they also found positive effect of MeJA on the mRNA transcription of *bAS* and saikosaponin production in another plant species, *Bupleurum falcatum* (Kim et al. 2011). Similarly, we found the relation between *CabAS* expression and asiaticoside production in centella cells treated with salicylic acid. Salicylic acid elicitation (50–150 µM) increased the mRNA transcription of *CabAS* in comparison with the wild type.

In centella cells, triterpene saponins and phytosterols are converted from precursor 2,3-oxidosqualene through two different pathways. The occurrence of these pathways is dependent on the activities of enzymes encoded by *CabAS* and *CaCYS* genes, respectively. Thus, the metabolic pathway relates to the expression levels of corresponding genes.

Kim et al. (2005c) reported that centella cells treated with MeJA inhibited the transcription of *CaCYS* while stimulating the synthesis of *CabAS* mRNA along with enhancing asiaticoside production. Mangas et al. (2008) have determined the centelloside content of calli grown in different culture media and analyzed the expression levels of some genes in the centelloside biosynthetic pathway. The results showed a low expression of the gene encoding β-amyrin synthase, and its centelloside content was <0.9 mg/g dry weight, while in the wild-type centella plants the centelloside content was from 1.5 to 2 mg/g dry weight.

In previous report, we found salicylic acid stimulated asiaticoside production (Loc and Giang 2012). This result is in accordance with the increasing expression level of *CabAS* as well as decreasing synthesis of *CaCYS* mRNA in present study (Fig. 2). Our results also confirmed that asiaticoside production is tightly regulated by the expression of both *CabAS* and *CaCYS* genes. Interestingly, the expression of *CaSQS* gene was inhibited by salicylic acid elicitation at concentrations from 50 to 200 µM. In contrast, Kim et al. (2005a) showed that *CaSQS* expression in centella cells was enhanced when MeJA is added to the culture medium. This observation is also found in *Medicago truncatula* (Suzuki et al. 2002), and *Glycyrrhiza glabra* (Hayashi et al. 1999). However, these studies were not performed in cell cultures. Thus, it seems that the regulation of *CaSQS* gene under elicitation of salicylic acid in centella cells differs from the wild type.

In other plant species such as *Linum album*, (Yousefzadi et al. 2010) found the cells treated with 10 μM salicylic acid for 3 days have increased podophyllotoxin production (PTOX) over three times that of the control. Also qPCR analyses showed that the expression of the genes coding for cinnamoyl-CoA reductase, phenylalanine ammonia-lyase and cinnamyl-alcohol dehydrogenase, all involved in the first steps of the PTOX biosynthesis, increased in salicylic acid-treated cells reaching a highest level after 8–12 h of the treatment. However, the expression of pinoresinol–lariciresinol reductase gene in the last steps of the PTOX biosynthesis was not affected by salicylic acid. According to Jumali et al. (2011), treatment with high concentration of salicylic acid is able to increase plant defense mechanism which later will induce the expression of genes encoding the biosynthesis of secondary metabolites in *Mitragyna speciosa*. The previous study of Wen et al. (2005) also showed that salicylic acid could increase the mRNA transcription of phenylalanine ammonia-lyase (PAL) and the biosynthesis of new PAL protein, and increase the activity in grape berry (*Vitis vinifera* L. cv. Cabernet Sauvignon).

In conclusion, the effect of salicylic acid on regulation of gene expression in the isoprenoid pathway can be applied in biotechnological production of centelloside and our results provide a suitable alternative to improve the centelloside biosynthesis in centella cells.
